# Oral Administration of Linoleic Acid Induces New Vessel Formation and Improves Skin Wound Healing in Diabetic Rats

**DOI:** 10.1371/journal.pone.0165115

**Published:** 2016-10-20

**Authors:** Hosana G. Rodrigues, Marco A. R. Vinolo, Fabio T. Sato, Juliana Magdalon, Carolina M. C. Kuhl, Ana S. Yamagata, Ana Flávia M. Pessoa, Gabriella Malheiros, Marinilce F. dos Santos, Camila Lima, Sandra H. Farsky, Niels O. S. Camara, Maria R. Williner, Claudio A. Bernal, Philip C. Calder, Rui Curi

**Affiliations:** 1 School of Applied Sciences, University of Campinas, Limeira, Brazil; 2 Department of Physiology and Biophysics, Institute of Biomedical Sciences, Sao Paulo University, Sao Paulo, Brazil; 3 Department of Genetics, Evolution and Bioagents, Institute of Biology, University of Campinas, Campinas, Brazil; 4 Cell and Developmental Biology Department, Institute of Biomedical Sciences, Sao Paulo University, Sao Paulo, Brazil; 5 Department of Clinical and Toxicology Analyses, School of Pharmaceutical Sciences, Sao Paulo University, Sao Paulo, Brazil; 6 Department of Immunology, Institute of Biomedical Sciences, Sao Paulo University, Sao Paulo, Brazil; 7 Food Sciences and Nutrition, School of Biochemistry and Biological Sciences, National University of Litoral, Santa Fé, Argentina; 8 Human Development and Health Academic Unit, Faculty of Medicine, University of Southampton, Southampton, United Kingdom; 9 NIHR Southampton Biomedical Research Centre, University Hospital Southampton NHS Foundation Trust and University of Southampton, Southampton, United Kingdom; Cedars-Sinai Medical Center, UNITED STATES

## Abstract

**Introduction:**

Impaired wound healing has been widely reported in diabetes. Linoleic acid (LA) accelerates the skin wound healing process in non-diabetic rats. However, LA has not been tested in diabetic animals.

**Objectives:**

We investigated whether oral administration of pure LA improves wound healing in streptozotocin-induced diabetic rats.

**Methods:**

Dorsal wounds were induced in streptozotocin-induced type-1 diabetic rats treated or not with LA (0.22 g/kg b.w.) for 10 days. Wound closure was daily assessed for two weeks. Wound tissues were collected at specific time-points and used to measure fatty acid composition, and contents of cytokines, growth factors and eicosanoids. Histological and qPCR analyses were employed to examine the dynamics of cell migration during the healing process.

**Results:**

LA reduced the wound area 14 days after wound induction. LA also increased the concentrations of cytokine-induced neutrophil chemotaxis (CINC-2αβ), tumor necrosis factor-α (TNF-α) and leukotriene B_4_ (LTB_4_), and reduced the expression of macrophage chemoattractant protein-1 (MCP-1) and macrophage inflammatory protein-1 (MIP-1). These results together with the histological analysis, which showed accumulation of leukocytes in the wound early in the healing process, indicate that LA brought forward the inflammatory phase and improved wound healing in diabetic rats. Angiogenesis was induced by LA through elevation in tissue content of key mediators of this process: vascular-endothelial growth factor (VEGF) and angiopoietin-2 (ANGPT-2).

**Conclusions:**

Oral administration of LA hastened wound closure in diabetic rats by improving the inflammatory phase and angiogenesis.

## Introduction

Wound healing is a physiological and essential process that must initiate as soon as tissue damage occurs. It is divided into 4 phases: 1) the formation of a clot, to stop the bleeding; 2) the inflammatory phase, with the recruitment of immune cells and release of inflammatory mediators; 3) the proliferative phase, with formation of granulation tissue, that plays an important role in new vessel formation; 4) the remodeling phase, when the spatial reorganization of collagen fibers and re-epithelization occur. Various cell types including neutrophils, macrophages, fibroblasts, endothelial cells and keratinocytes, and a great number of mediators (e.g. cytokines, lipid derived molecules, growth factors) orchestrate the wound healing phases. Alterations in duration or intensity of the inflammatory phase modify the onset of the next phase and hence impair the wound healing process [1, 2].

Types 1 and 2 diabetes exhibit different etiologies, however, both are associated with hyperglycemia and impairment in wound healing through mechanisms involving exacerbation and chronification of the inflammatory response [2–4]. Hard-to-heal wounds are a well-known diabetic complication [5]; 25% of diabetic patients had experienced a non-healing ulcer and 28% of them underwent amputation related to poor wound healing [5]. Chronic wounds have an imbalanced production of pro- and anti-inflammatory mediators such as TNF-α, IL-1β, VEGF and IL-10 [6–8], hindering proper healing. The sustained expression of pro-inflammatory cytokines and chemokines are associated with increased numbers of neutrophils in late wound tissues and impairment in tissue repair in db/db mice [4]. The recruitment of macrophages is also impaired and there is a predominance of M1 pro-inflammatory macrophage subtype in the harmed area. The permanence of M1 macrophages in wound tissue increases the production of inflammatory mediators and blocks inflammation resolution. As a consequence, the progression to angiogenesis not occurs [3, 9].

Angiogenesis is defined as the formation of new vessels from preexisting vessels [10]. It plays a critical role in wound healing, since it reestablishes the supply of oxygen and nutrients to damaged area as well as promotes the migration of cells that will build up the tissue. Angiogenesis is up regulated by growth factors such as VEGF and ANGPT-2, that will promote the genesis of new vessels by acting on endothelial cells [11]. On the other hand, it is down regulated by angiostatin and TGF-β (tumor growth factor-β) that, not only, reduce the synthesis of pro-angiogenic factors but also antagonize some of their effects [12]. Then, both inflammation and angiogenesis play pivotal roles in injured tissue repair. These two processes are impaired in diabetes, resulting in delayed wound healing. Compounds that reestablish inflammation and angiogenesis and then normalize the wound healing process are of great importance for diabetic patients.

Skin wounds are popularly treated with natural compounds such as nut oils in developping countries. Although this provides the basis for the pharmaceutical formulations of healing ointments, little is known about how these products act on the wound healing process. We previously reported that oral administration of pure linoleic acid (LA), an abundant fatty acid of nut oils, improves the wound healing process in non-diabetic animals [13]. LA (18:2, ω-6) is an essential fatty acid widely present in the western diet. LA constitutes 40% of the fatty acids in the human skin and plays an important role for its function. However, there is no consense about the effects of LA on inflammatory response yet. We reported that oral administration of LA has pro- and anti-inflammatory effects in non-diabetic rats. LA increased the influx of inflammatory cells into the injured tissue, changed neutrophil [14] and macrophage [15] fatty acid composition, and reduced the production of cytokines and reactive oxygen species (ROS).

The information above led us to investigate the effects of oral administration of LA on skin repair in diabetic rats. The key steps of wound healing, inflammation and angiogenesis, were assessed. We hypothesized that LA may hasten wound healing by acting on inflammatory response and angiogenesis. To test this hypothesis the experiments were performed *in vivo* in streptozotocin-induced diabetic rats orally suplemented with pure LA.

## Materials and methods

### Animals

Male Wistar rats (from the Institute of Biomedical Sciences, Sao Paulo University, Brazil) were maintained at 23°C under a light: dark cycle of 12:12 h and received food (Nuvital, Curitiba, Brazil, containing 22% of protein, 4,5% of fat, 40,8% of carbohydrate and 8% of fiber) and water *ad libitum*. Linoleic acid constitutes 40% of the fatty acids in the chow. The complete fatty acid composition of chow was previously published [14]. The Animal Care Committee of the Institute of Biomedical Sciences approved the experimental procedure of this study (Protocol number: 86).

### Induction of diabetes mellitus

**Fig 1:**
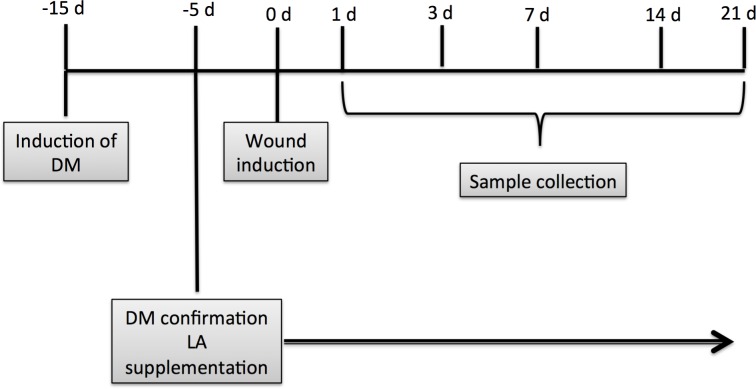
Experimental protocol.

Type I diabetes *mellitus* was induced by streptozotocin injection as previously reported [16]. This drug destroys pancreatic beta cells resulting in a marked reduction in insulin release and consequently hyperglycemia. Diabetes was confirmed three days after induction by blood glucose concentrations above 250 mg/dL as determined by the Accu-Check Active glucometer (Roche, Mannheim, Germany). After ten days, diabetic animals were divided into two groups: untreated diabetic (D) and diabetic that received oral LA supplementation (DLA) (Fig 1).

### Administration of LA

Oral administration of pure LA (Sigma-Aldrich Co, St Louis, MO, USA) was initiated ten days after diabetes induction and maintained daily during the experimental period. Unesterified LA, at a dose of 0.22 g/kg b.w, was administered by gavage. The total calories associated with LA dose are low (1.98 cal/day) and so, we used water administration as control [14]. The D group received water (same LA volume). Considering the chow FA composition [14] and the food intake (g/day) of the animals (data not shown), the LA dose used in the present study represents an increase of 7% in the total ingestion of LA compared with the chow diet.

### Skin wound induction

After five days of LA administration, the animals were anesthetized with xilazine (7 mg/kg b.w.) and ketamine (14 mg/kg b.w.) and an area of 10 mm^2^ in dorsal region skin was shaved and removed by surgery. Animals were killed by overdose of the anesthetics xilazine (21 mg/kg b.w.) and ketamine (42 mg/kg b.w.), 1, 3, 7 or 14 days after the surgery.

### Determination of wound tissue fatty acid composition

The fatty acid composition of the wounds was determined by gas chromatography (GC) as previously described [17]. Results of individual fatty acids are expressed as percentage of total fatty acids.

### Skin wound closure assessment

Animals were anesthetized with isoflurane. The wounds were daily photographed using a Sony cyber shot camera (model DSC-S950S 10 mP; 4 x Optical zoom) by the same examiner, as previously described [13]. Wound closure was defined as a reduction of wound area and results are expressed as percentage of the original wound area.

### Eicosanoid measurements in wound tissue

The concentrations of leukotriene B_4_ (LTB_4_) and 15 (S) hydroxyeicosatetraenoic acid (15(S)-HETE) were measured in scar tissue homogenates using ELISA kits according to manufacturer's instructions (Cayman Chemical, Ann Arbor, MI, USA).

### Histological examination of the wound tissue

Wound lesions with adjacent normal skin were removed, fixed in Bouin for 24 h at room temperature, processed and embedded in Paraplast®. Seven μm sections were stained with hematoxilin/eosin to evaluate the general morphology of the wound.

### Morphometric analysis of blood vessels

Digital photomicrographs were obtained using a Leitz Aristoplan optical microscope (Leica) with a 20x objective and a Nikon (DS-Ril) camera. The NIS-Elements software was employed for image capturing. Only the dermal wound region, just below the crust, was photographed (2–5 pictures per animal, 3–4 animals per group). The Image J public software (NIH, Bethesda, US) was used for morphometric analysis using the grid plugin. A grid of 130 points was used in each photograph and the number of points observed in the interior of small blood vessels was counted and expressed as percentage of the total points, representing the area occupied by vessels.

### Cytokine contents in wound tissue

Wound lesions removed at 1, 3 and 7 days after lesion induction were wrapped up in aluminium paper, dropped into dry ice and kept frozen (-80°C). CINC-2αβ, IL-1β, TNF-α, IL-6 and VEGF were assessed by ELISA as previously described [13] using the Duo Set kit (R&D System, Minneapolis, MN, USA) and normalized by protein concentration as measured by the Bradford method [18].

### Real-time polymerase chain reaction

Total RNA was extracted (RNAeasy Mini Kit, Qiagen, Venlo, Netherlands) from wound tissue and reverse-transcribed using the High-Capacity cDNA Reverse Transcription kit (Applied Biosystems, Foster City, CA, USA). Reactions were performed using SYBR-Green PCR master mix (Invitrogen, Carlsbad, CA, USA) in a Rotor Gene Q (Qiagen, Germantown, Maryland, MD, USA). mRNA expression was normalized by the D values in unwounded skin. The sequences of the primers used are described in the S1 Table.

### Measurement of NF-KB and AP-1 activation in wound tissue

Wound tissue removed at 1 and 24h after lesion was processed as previously described [13, 19]. The blots were analyzed by scanner densitometry (Image Master 1D, Amersham Biosciences) and results expressed as arbitrary units in relation to diabetic animals.

### Statistical Analysis

Comparisons between groups were performed using Student’s t test. In some experiments (cytokines, skin fatty acid composition and mRNA expression), two-way analysis of variance (ANOVA) and Bonferroni post-test were used. The significance was set at p<0.05.

## Results

All streptozotocin-induced diabetic animals used in this study had blood glucose levels close to 400 mg/dL. None other plasma measurement (e.g. ketone bodies) was considered for this purpose as also reported by others [16, 20, 21]. The diabetes protocol used was established considering the animals lost around 10% of their body weight and they would not survive for longer period without insulin administration. The diabetic rats were not treated with insulin due to its direct effects on the wound healing process [20]. The combination of high glycemia, intense weight loss and general catabolic state could compromise the interpretation of results obtained in a condition of prolonged diabetes state. Ten days after streptozotocin-induced diabetes, a full-thickness biopsy was performed and wound closure was assessed over time. The diabetic condition protocol used did delay wound healing as indicated by the analysis of wound closure in control (non-diabetic) and diabetic (non-treated) rats.

Pure LA was orally administered to diabetic rats daily for 5 days prior to the full-thickness biopsy and then until wound closure (Fig 1). The dose (0.22 g/kg/day) of LA and the duration of the administration did not induce any change in the nutritional status of the animals (data not shown). The amount of LA given is unlikely to have increased plasma ketone body levels. In fact, the dose of LA given represents an increase of 7% in the total ingestion of LA compared with the chow diet.

### Oral administration of LA changed skin fatty acid composition and modulated eicosanoid production in wound tissue

**Fig 2:**
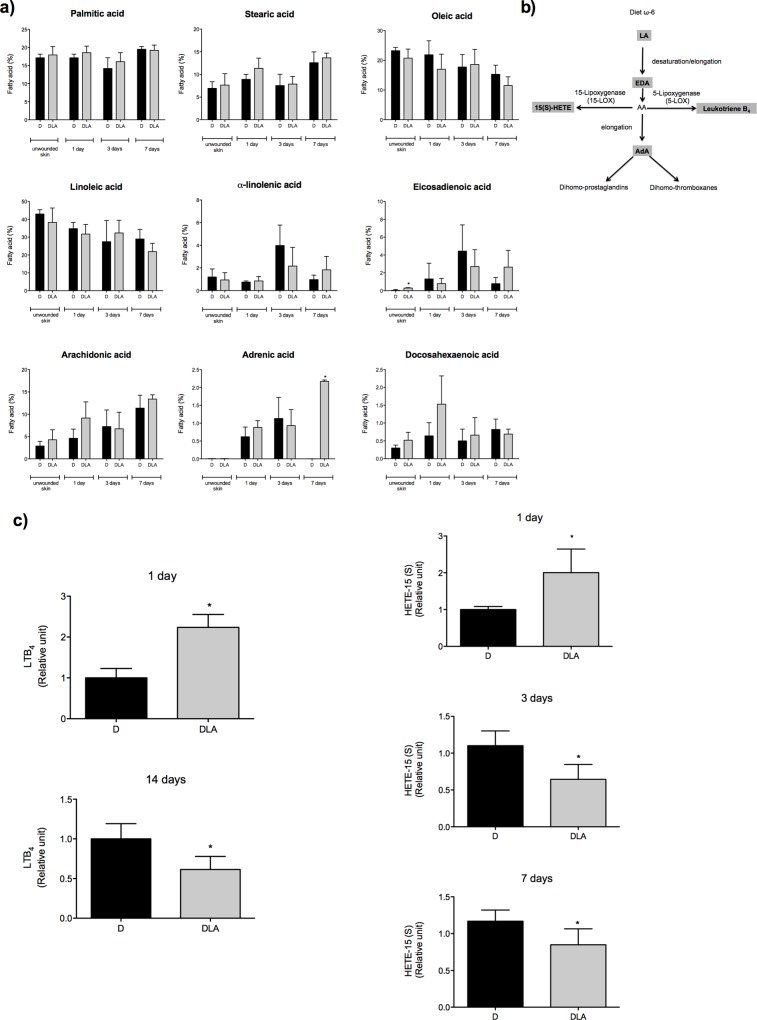
Fatty acid composition and eicosanoids production during wound healing. **(a)** Fatty acid composition in wound tissue from diabetic rats (D) and diabetic rats treated with linoleic acid (DLA). Results are presented as mean ± SD. D (3 rats) and DLA (7 rats). (*) Indicates significant differences between D and DLA rats (p<0.001). **(b)** Scheme showing LA metabolism and generation of eicosanoids. **(c)** LTB_4_ and HETE-15 (S) concentrations in wound tissues from diabetic rats (D) and diabetic rats treated with linoleic acid (DLA). Results are presented as mean ± SD. D (3 rats) and DLA (5 rats). (*) Indicates significant differences between D and DLA rats (LTB_4_ 1d –p = 0.002; LTB_4_ 14d –p = 0.02; HETE-15 (S) 1d –p = 0.03; HETE-15 (S) 3d –p = 0.001; HETE-15 (S) 7d –p = 0.04).

We previously reported that the same treatment protocol increases the proportion of LA in neutrophils [14] and macrophages [15]. LA increased eicosadienoic (EDA) percentages on unwounded skin. On the 7^th^ day, LA elevated the adrenic acid (AdA) percentages in wound tissue (Fig 2A).

The concentrations of LTB_4_ and 15(S)-HETE, two eicosanoids derived from AA, which can be generated from LA (Fig 2B) were measured. Concentrations of both eicosanoids were increased in the wound tissue one-day post-wounding and were reduced after 3 and 7 days (15(S)-HETE) or 14 days (LTB_4_) in the DLA group (Fig 2C).

### LA improved skin repair in diabetic rats

**Fig 3:**
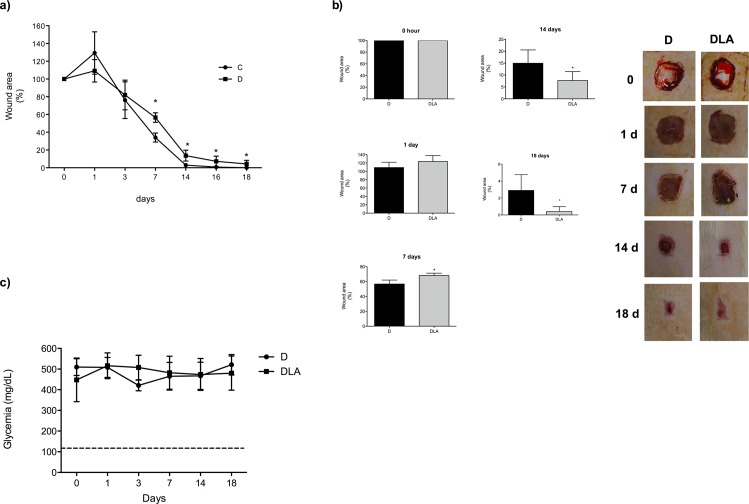
Time course of wound healing and glycemia. **(a)** Macroscopic and time course of wound closure in control (C) and diabetic rats (D). (*) Indicates significant differences among C versus D (p = 0.006) **(b)** Macroscopic and time course of wound closure in diabetic (D) and diabetic rats treated with LA (DLA) (*) Indicates significant differences between D and DLA (p = 0.02). Representative photos of the wound tissue obtained during the time-course of 18 days. Results are presented as mean ± SD. D (5 rats) and DLA (9 rats). **(c)** Glycemia of rats during the wound healing process: (D) diabetic; (DLA) diabetic rats treated with LA. Dashed line indicates the mean of glycemia in control rats.

Fourteen days post-wounding, the original wound area was reduced by over 95% in the control group being fully closed by day 18 (Fig 3A). In comparison, wound closure was much slower in diabetic animals. At the 7^th^ day after wound induction, diabetic animals had a larger wound area (p = 0.002) than the control group (D: 56 ± 2% vs. C: 34 ± 3%, mean ± SEM of 5–9 animals per group) (Fig 3A). The delay in wound healing remained in diabetic animals and wounds were not fully healed up to 18 days after induction.

Administration of LA hastened wound closure in diabetic rats (Fig 3B), an effect that was independent of any change in glycemia (Fig 3C). The wound area was reduced in the DLA group from the 14^th^ to the 18^th^ day post-wounding in relation to D animals (Fig 3B). In order to verify if the effect on wound closure was specific for LA, we performed the same analysis in diabetic rats treated with pure oleic acid (OA), a monounsaturated 18-carbon chain fatty acid (S1 Fig). In contrast to the effect of LA, OA caused a delay in the wound closure of diabetic rats (DOA group) when compared to D animals but did not modify glycemia (S1 Fig). Taking together, these results suggest that the improvement in wound healing is specific for LA treatment.

### LA induced inflammatory cell migration and increased formation of new vessels in wound tissue

**Fig 4:**
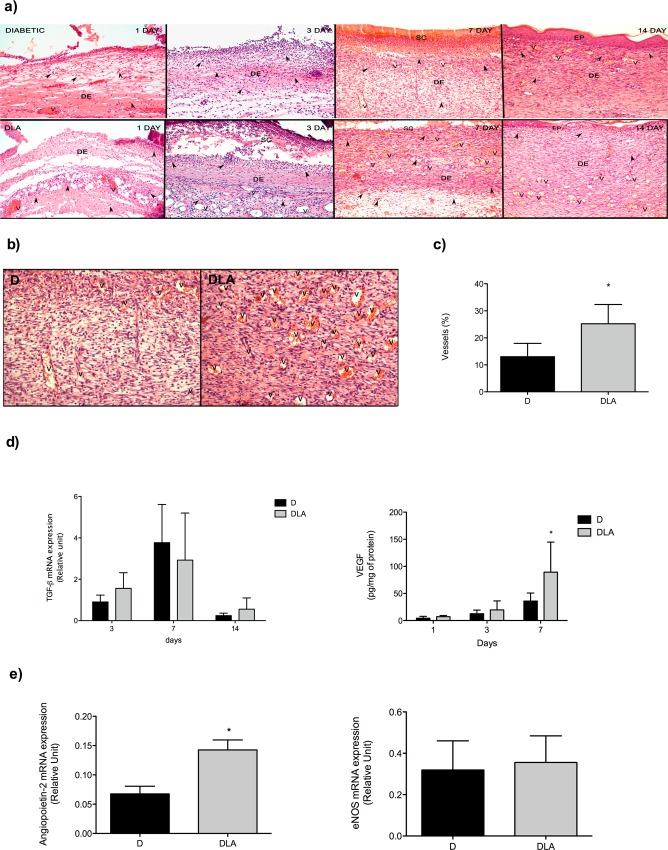
Histological analysis and angiogenic growth factors expression in wound tissue. **(a)** Samples were isolated from diabetic rats (D) and diabetic rats treated with linoleic acid (DLA) at the 1^st^, 3^rd^, 7^th^ and 14^th^ days after wounding. **(b)** Representative new vessel formation in wound tissue from the D and DLA groups. Samples were collected on the 7^th^ day after wounding. **(c)** Vessels quantification. Results are presented as mean ± SD. D (4 rats) and DLA (5 rats). (*) Indicates significant difference between D and DLA (p = 0.0001). **(d)** TGF-β mRNA expression and VEGF concentration in wound tissues from diabetic rats (D) and diabetic rats treated with linoleic acid (DLA). Results are presented as mean ± SD. D (9 rats) and DLA (4 rats). (*) Indicates significant difference between D and DLA (VEGF–p<0.01). **(e)** eNOS and ANGPT-2 mRNA expression in wound tissues from diabetic rats (D) and diabetic rats treated with linoleic acid (DLA). Results are presented as mean ± SD. D (9 rats) and DLA (4 rats). (*) Indicates significant difference between D and DLA (ANGPT-2 –p = 0.01). V: vessel. DE: derm. Objective 10X.

Histological analysis of wounds from diabetic rats exhibited inflammation in the dermis on the first day and intense neutrophil influx into the tissue from the 3^rd^ until to the 14^th^ day post-wounding (Fig 4A). A few vessels were observed in wounds of diabetic rats (Fig 4A).

Wounds were more inflamed in DLA group than in D animals on the first day after wounding. Significant edema and high number of neutrophils were found in the crust (Fig 4A). On the 3^rd^ day, neutrophils were abundant at the surface of the wound but in lower number than in the D group. There were more newly formed vessels from the 3^rd^ day until the 14^th^ day after wounding in the DLA group in relation to D animals (Fig 4B and 4C).

To explain the increase in vessel number observed in the DLA group, we measured mRNA expression of tissue factors that regulate angiogenesis. Although there was no difference in TGF-β expression, the concentration of VEGF was elevated in DLA rats (Fig 4D), 7 days after wound induction. Considering this effect on VEGF, we analysed the expression of other pro-angiogênic factors at the 7^th^ day after tissue injury and observed that DLA increased ANGPT-2 mRNA expression but did not alter eNOS (endothelial nitric oxide synthase) expression (Fig 4E). These effects of LA were in agreement with the presence of new vessels observed in the histological analysis (Fig 4A, 4B and 4C). Thus, LA induced migration of inflammatory cells and increased the formation of new vessels in wound tissue.

### LA affected both the early and the late cell recruitment

**Fig 5:**
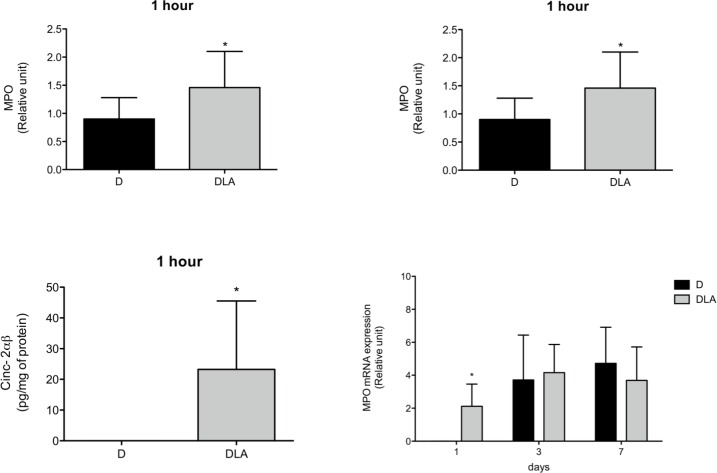
Myeloperoxidase and CINC-2αβ contents. Myeloperoxidase (MPO) activity (1 hour), mRNA expression (1 h, 1, 3 and 7 days) and CINC-2αβ concentration (1 h) in wound tissue. Results are presented as mean ± SD. D (6 rats) and DLA (6 rats). (*) Indicates significant differences between D and DLA rats (MPO activity–p = 0.02; mRNA expression 1h 0.006; mRNA 1 day–p = 0.03; CINC-2αβ –p = 0.04).

In order to evaluate the kinetics of inflammatory cell migration into wound tissues, mRNA expression of neutrophil (myeloperoxidase—MPO) and macrophage (F4/80) markers were measured during the wound healing process. MPO activity and chemokine concentrations were also measured at different time points in the wound tissue. LA increased MPO mRNA expression and activity one hour after wound induction. This was followed by elevation in CINC-2αβ, an important neutrophil chemoattractant agent (Fig 5). The increase in MPO mRNA expression persisted until the 1^st^ day after wound induction.

**Fig 6:**
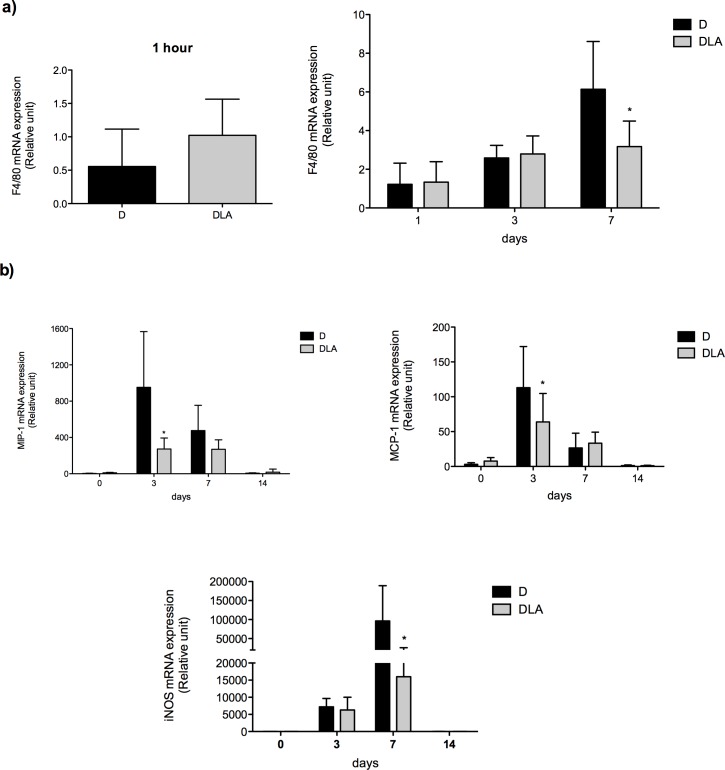
Macrophages cell markers expression. **(a)** mRNA expression of F4/80 in wound tissue 1 hour and 1–7 days after wounding. Results are presented as mean ± SD. D (5 rats) and DLA (10 rats). **(b)** mRNA expression of MIP-1, MCP-1 and iNOS in wound tissue from diabetic rats (D) and diabetic rats treated with linoleic acid (DLA). Results are presented mean ± SD. D (5 animals) and DLA (9 animals). (*) Indicates significant differences between D and DLA rats (p<0.001)

After neutrophils, the next cell population that migrates into an injured area is macrophage. LA did not change F4/80 (macrophage marker) expression during the inflammatory phase of wound healing (Fig 6A). However, LA diminished it at 7^th^ day. This result was followed by reduction in the contents of chemoattractant cytokines (MIP-1 and MCP-1) and of iNOS expression (Fig 6B) that is increased in activated macrophage [22]. So, LA treatment accelerated the early migration of neutrophils through, at least in part, an increase in production of chemoattractants or neutrophil responsiveness to them. LA also modified migration of macrophages (later) and production of macrophage-related chemoattractants.

### LA hastened the inflammatory phase

**Fig 7:**
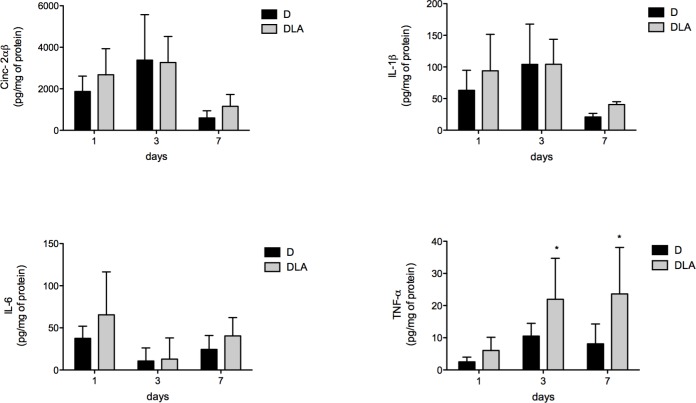
Cytokines production during wound healing. CINC-2α, IL-6, IL-1β and TNF-α concentrations in wound tissue from diabetic rats (D) and diabetic rats treated with linoleic acid (DLA). Results are presented mean ± SD. D (5 animals) and DLA (6 animals). (*) Indicates significant differences between D and DLA rats (TNF-α 3d –p<0,05; TNF-α 7d –p<0.01)

**Fig 8:**
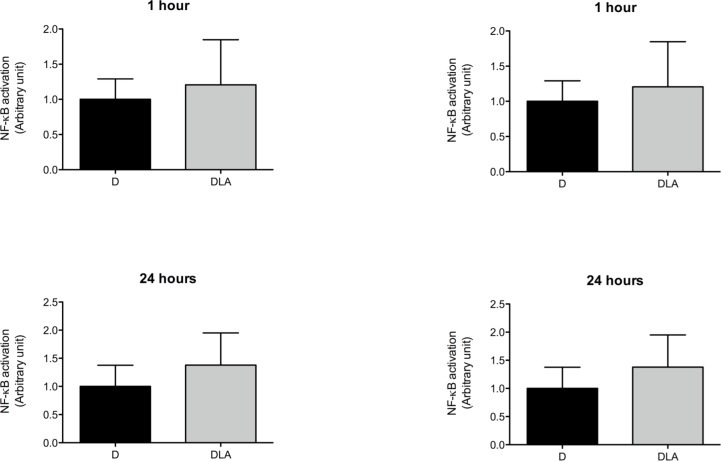
Transcription factors activation. NF-KB and AP-1 activation in wound tissues from diabetic rats (D) and diabetic rats treated with linoleic acid (DLA). Results are presented as mean ± SD. D (5 animals) and DLA (8 animals). (*) Indicates significant difference between D and DLA rats (AP-1 1 h–p = 0.02; AP-1 24hs–p = 0.001)

TNF-α concentration was raised in wounds on days 3 and 7 after lesion in the DLA group in comparison to D rats (Fig 7). We did not observe any change in IL-6 or IL-1β levels between the experimental groups (Fig 7). We also evaluated activation of NF-κB and AP-1 in the wound tissue. No alteration was observed in NF-κB activation. However, LA inhibited AP-1 activation 1 and 24 hours after wound induction in diabetic animals (Fig 8).

Oral administration of LA hastened wound healing inflammation and angiogenesis steps in diabetic rats by: 1) increasing inflammatory cell influx through chemoattractant agent (CINC-2αβ) production and LTB_4_ generation; 2) regulation in gene expression (MIP, MCP and iNOS), through AP-1 modulation; 3) induction of vessel formation via production of pro-angiogenic factors (ANGPT-2 and VEGF).

## Discussion

The animals herein used had a glycemia around 400 mg/dL (not affected by the treatment with LA or wound process). Despite the short period of diabetes impaired in wound healing was reported, in comparison to non-diabetic animals, which resembles the human condition. Oral administration of LA to diabetic rats hastened the influx of neutrophils (early), reduced macrophage (late) abundance, and modulated the production/release of cytokines (CINC-2αβ and TNF-α), growth factors (VEGF) and eicosanoids (LTB_4_ and 15(S)-HETE) that drive the healing process. These modifications in LA treated rats were associated with new vessel formation and improvement of the wound healing process.

In order to examine if the effects of LA on wound healing process were due to LA incorporation in the skin, we evaluated skin fatty acid composition by gas chromatography (GC). Although no differences were observed in LA or AA incorporation, oral administration of LA increased eicosadienoic (EDA– 20:2 ω-6) and adrenic acid (AdA—22:4ω-6) incorporation (Fig 2A). EDA is a product of LA elongation that also modifies the inflammatory response, however, in a less intense manner when compared to LA or AA [23]. AdA is an AA elongation product, which can be metabolized to dihomo-eicosanoids or docosanoids [24, 25]. A reduction in AdA formation has been described in type 1-diabetes [26]. Importantly, AdA reduces AA metabolism and inhibits AA-derived eicosanoid formations [27]. In the present study, LA increased AdA incorporation and reduced 15(S)-HETE (Fig 2C).

15-HETE plays a key role in the early phase of wound healing since it controls clot formation through platelet aggregation and thrombin production [28]. Long standing release of 15-HETE is positively associated with wound tissue infiltration of neutrophils and macrophages [29]. The presence of 15-HETE in the latter phase of wound healing reflects a persistent influx of inflammatory cells into the tissue and consequently wound chronification.

Fatty acids can generate a wide range of bioactive molecules named oxylipins [30]. Oxylipins are products formed by PUFA oxidation and the most well known class is the AA-derived eicosanoids [30]. However, they can also be derived from LA such as 13 hydroxyoctadecadienoic acid (13-HODE) and 9,10-cis epoxide of linoleic acid (9,10 EpOME). Considering that HODE and EpOME oxilipins can modulate inflammatory responses, a limitation of the present study is the fact we did not measure these molecules during the wound healing process.

Diabetes *mellitus* is associated with chronic inflammation and poor wound healing [31]. The inflammatory phase of wound healing in diabetes exhibits accumulation and persistence of primed inflammatory cells in the lesion area [32], resulting in exacerbated production of pro-inflammatory mediators that cause surrounding tissue damage and impairs wound resolution [3, 31, 33].

During inflammation, leukocyte recruitment cascade, a sequential adhesive interaction between leukocytes and endothelial cells, takes place [34]. LA administration induced neutrophil infiltration in the first hours after wounding that returned to basal values 3 days latter. The possible mechanisms involved in LA-induced cell migration are: increased adhesion molecule expression in leukocytes [14] and endothelium [35] and release of chemoattractants such as MCP, LTB_4_ and CINC-2αβ [36]. The earlier expression of CINC-2αβ induced by LA, also reported in the present study (Fig 5), is associated with an increase in neutrophil influx into damaged tissue and with acceleration of colonic wound healing [37]. Once in the injured area, neutrophils phagocyte dead cells and microorganisms and produce cytokines that attract macrophages to wounded site.

Macrophages modify their phenotype in response to the wound environment. Due to their plasticity, different states of polarization were described for these cells, in which M1 (pro-inflammatory) and M2 (pro-resolution) are the extremes [38]. In a short time wound healing, the switch of M1 to M2 macrophages hastens the resolution of inflammation enabling the progression to the proliferative phase [39]. On the other hand, in chronic wounds, the persistence of M1 macrophages in the tissue exacerbates the inflammatory response and blocks the progression to wound resolution [3].

Considering the importance of macrophages on wound healing, we investigated if LA could influence their recruitment to the wound area. Although we did not analyze M1/M2 markers, we found that LA diminished the expression of a global macrophage marker (F4/80) and reduced the production macrophage derived chemokines (MCP-1 and MIP-1) in the late inflammatory phase (7 days). MCP-1 is a chemokine produced by several cell types including keratinocytes, endothelial cells and resident macrophages, which induces migration of inflammatory cells to injured tissue. Maximum expression of MCP-1 occurs 1–2 days after wounding and declines progressively until to the 7^th^ day of the wound healing process in control conditions [40].

We have previously demonstrated that LA induces transient AP-1 activation in skin of non-diabetic rat [13], favoring the recruitment and activation of inflammatory cells. The effect was not found herein in diabetic rat. LA reduced AP-1 activation at 1 and 24 hours after wounding. Neub et al. [41] stated that reduction in AP-1 activity is needed to restore normal wound healing and prolonged AP-1 activation is described in chronic wounds [42]. The modulation of AP-1 activation in skin is shared by other fatty acids such as docosahexaenoic acid (DHA) and by eicosanoids such as 13-hydroxyoctadecadienoic acid (13-HODE) and 15-hydroxyeicosatrienoic acid (15-HETrE) [43]. These reports together indicate that LA modulates recruitment of cells through inhibition of AP-1 activity and consequent reduction on chemokine production.

After recruitment, leukocytes produce a range of inflammatory mediators such as cytokines, ROS, and growth factors to resolve inflammation. IL-6, IL-1β and TNF-α play a very important role in this process. IL-6 promotes the migratory response of epithelial cells [44] and wound remodeling [45]. IL-6 deficient mice exhibit impaired angiogenesis, macrophage infiltration and re-epithelization, resulting in delayed wound healing [46]. IL-1β inhibits type I collagen production and upregulates metalloproteinase-1, which degrades collagen fibers [47]. Collagen is the main component of the extracellular matrix and plays an important role in the wound healing remodeling [48]. The reduction in type I collagen induces premature collagen synthesis and poor healing.

TNF-α is an important regulator of cell migration. Naaldijk et al. [49] reported that the presence of TNF-α in the medium increases migration of mesenchymal stem cells in a transwell assay. The migratory cell response plays a critical role in the proliferative phase of wound healing. TNF-α also induces angiogenesis *in vivo* [50] and *in vitro* [51] through increased VEGF production. Increased TNF-α and VEGF production in diabetic animals treated with LA explains the augmented number of new vessels and improved healing. Increased VEGF levels and number of new vessels formed support the proposition that LA induces angiogenesis.

Angiogenesis is necessary to deliver immune cells, nutrients and oxygen and to remove debris from the damaged tissue. Impairment in formation of new blood vessels retards the healing process and induces ulceration [52]. There is a wide range of growth factors that regulate angiogenesis [52–54]. Diabetes *per se* leads to increased TGF-β expression during tissue repair. High levels of TGF-β increase extracellular matrix deposition that impairs the vascularization process [55, 56]. Geng et al. [55] reported that there is an inverse correlation between TGF-β expression and VEGF concentration in colon tumors. TGF-β reduces VEGF stability by inducing ubiquitination and degradation of this growth factor, with no effect on VEGF mRNA levels [55].

Growth factors and cytokines released during inflammation are involved in the abluminal sprouting and formation of new vessels from an existing vessel [57–59]. Nishioka et al. [60] described that *in vivo* administration of LA induces angiogenesis through angiostatin suppression. Angiostatin is a proteolytic fragment of plasminogen and suppresses angiogenesis by inhibiting endothelial cell proliferation and migration and by inducing endothelial cell apoptosis [61]. In the present study, we did not detect angiostatin mRNA expression seven days after the wound in any group (data not shown). However, LA increased VEGF production and expression of ANGPT-2. This latter protein is induced by growth factors such as VEGF after endothelial cell [11] and/or fibroblast/myofibroblast [62] activation. The presence of ANGPT-2 primes endothelial cells to respond to inflammatory cytokines, resulting in expression of adhesion molecules and transmigration of inflammatory cells [11]. LA increased the production of pro-angiogenic factors, which might be associated with elevation in vascularization. These effects of LA are in agreement with the presence of new vessels observed in the histological analysis (Fig 4A, 4B and 4C).

In summary, oral administration of LA to diabetic animals brought forward the inflammatory response and induced angiogenesis. The pro-healing effects of LA hasted the healing process in diabetic rats.

## Supporting Information

S1 Fig Time course of wound closure in diabetic (D) and diabetic treated with oleic acid (DOA) rats. Results are presented as mean ± SD of 7 animals in each group. (#) Indicates differences in relation to D (10d –p = 0.04; 16d –p = 0.03; 18d –p = 0.03). Glycemia of rats during the wound healing process: (D) diabetic; (DOA) diabetic rats treated with OA. Dashed line indicates the mean of glycemia in control rats.
(TIF)

S1 Table Primer sequences. (DOCX)
