# Correction: Oral Administration of Linoleic Acid Induces New Vessel Formation and Improves Skin Wound Healing in Diabetic Rats

**DOI:** 10.1371/journal.pone.0179071

**Published:** 2017-05-31

**Authors:** Hosana G. Rodrigues, Marco A. R. Vinolo, Fabio T. Sato, Juliana Magdalon, Carolina M. C. Kuhl, Ana S. Yamagata, Ana Flávia M. Pessoa, Gabriella Malheiros, Marinilce F. dos Santos, Camila Lima, Sandra H. Farsky, Niels O. S. Camara, Maria R. Williner, Claudio A. Bernal, Philip C. Calder, Rui Curi

Fig 5 and Fig 8 are incorrect. The authors have provided corrected versions here.

**Fig 5:**
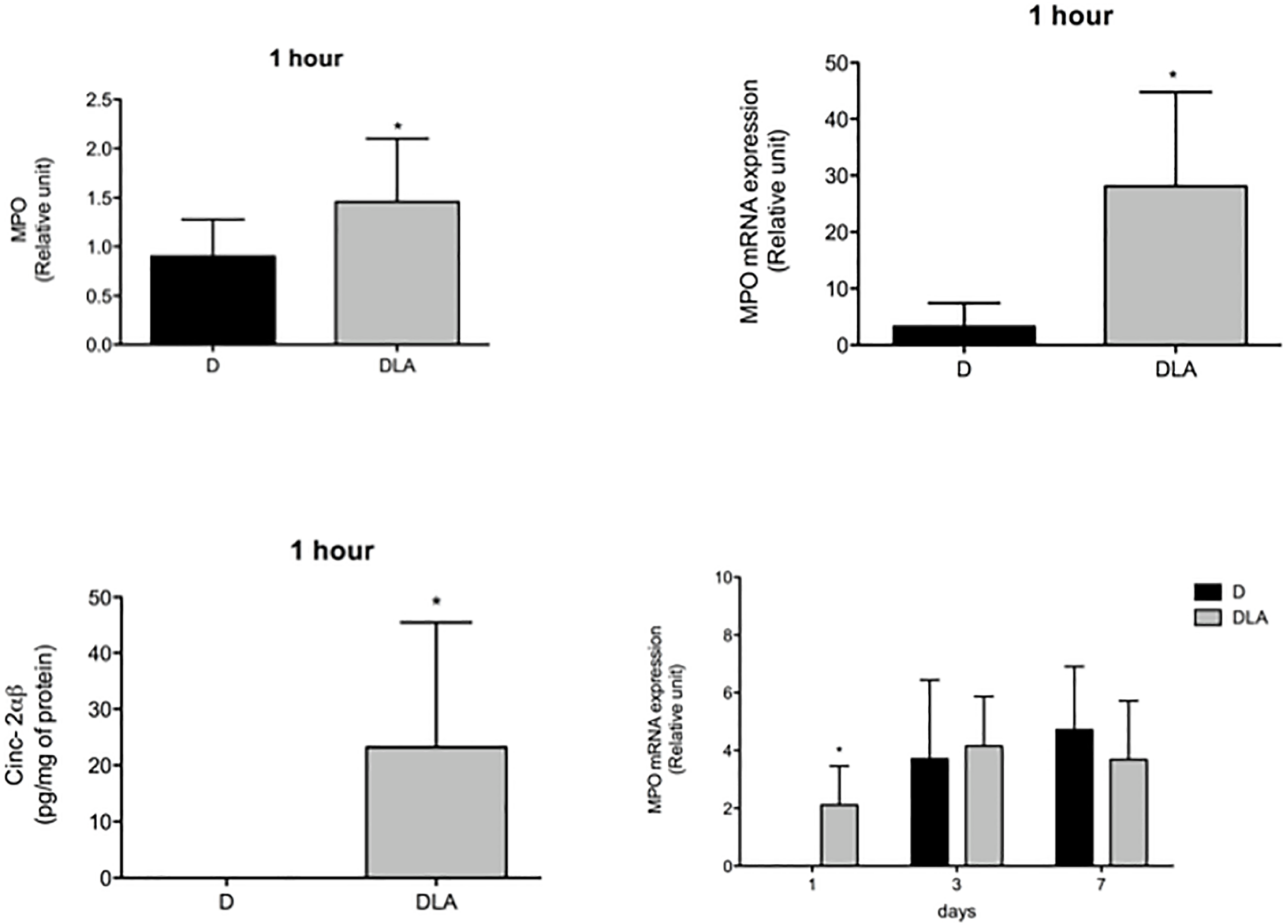
Myeloperoxidase and CINC-2αβ contents. Myeloperoxidase (MPO) activity (1 hour), mRNA expression (1 h, 1, 3 and 7 days) and CINC-2αβ concentration (1 h) in wound tissue. Results are presented as mean ± SD. D (6 rats) and DLA (6 rats). (*) Indicates significant differences between D and DLA rats (MPO activity–p = 0.02; mRNA expression 1h 0.006; mRNA 1 day–p = 0.03; CINC-2αβ –p = 0.04).

**Fig 8:**
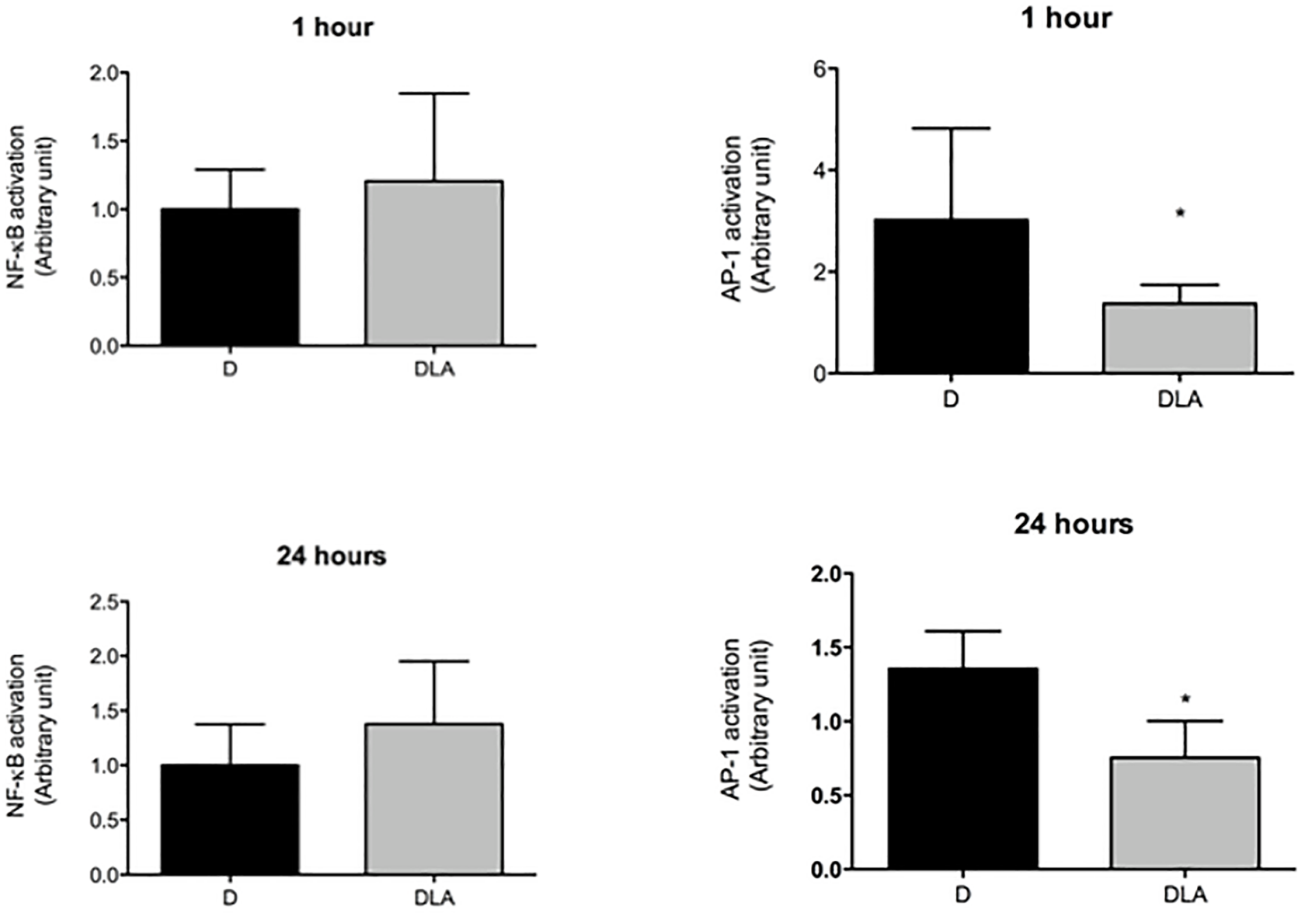
Transcription factors activation. NF-KB and AP-1 activation in wound tissues from diabetic rats (D) and diabetic rats treated with linoleic acid (DLA). Results are presented as mean ± SD. D (5 animals) and DLA (8 animals). (*) Indicates significant difference between D and DLA rats (AP-1 1 h–p = 0.02; AP-1 24hs–p = 0.001).
