# Supplementary figures and images for: Oral Administration of Linoleic Acid Induces New Vessel Formation and Improves Skin Wound Healing in Diabetic Rats
Source: PLoS One. 2016 Oct 20;11(10):e0165115. doi: 10.1371/journal.pone.0165115 (PMC5072690; doi:10.1371/journal.pone.0165115)

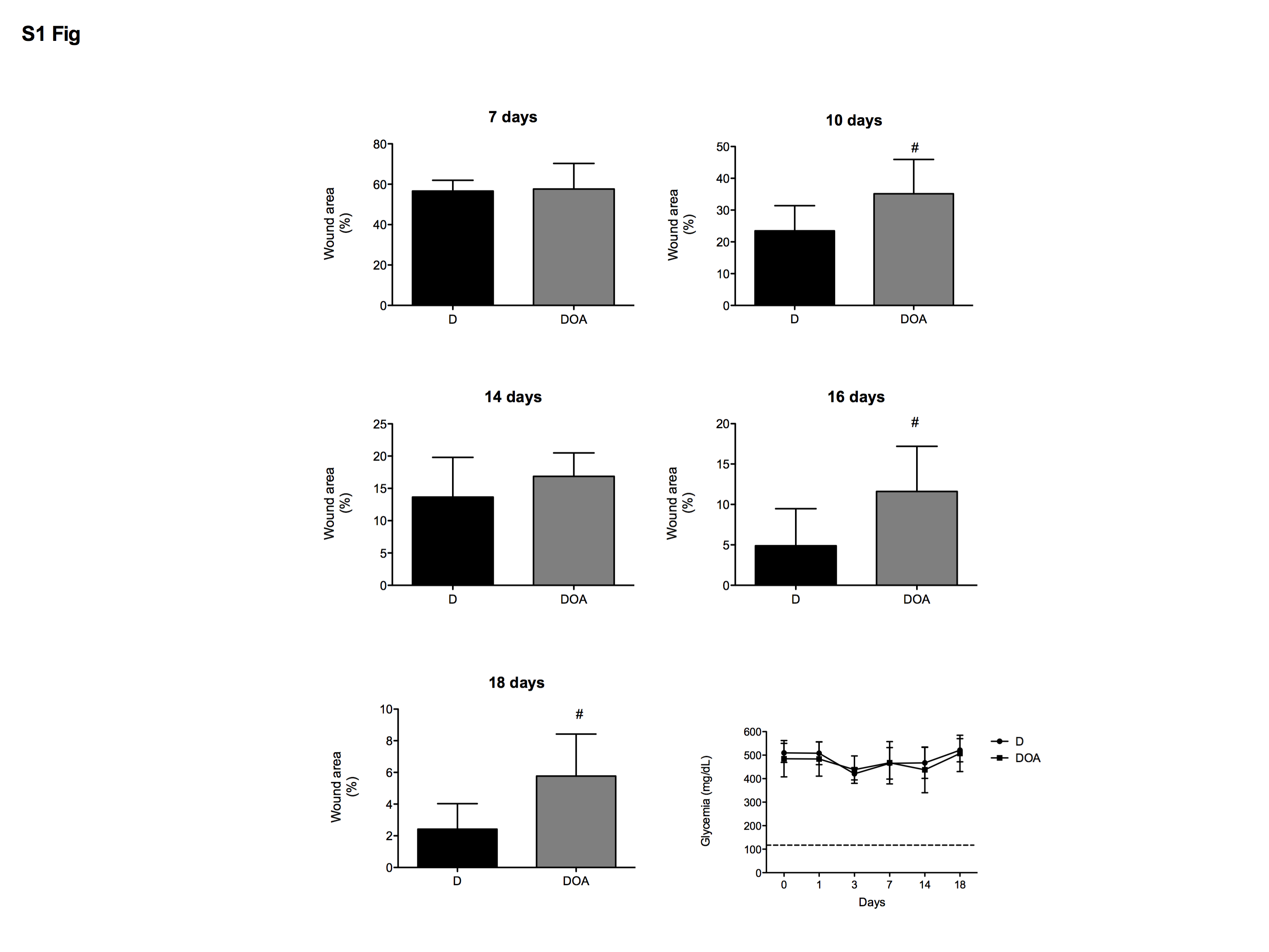

Supplement: S1 Fig — Results are presented as mean ± SD of 7 animals in each group. (#) Indicates differences in relation to D (10d –p = 0.04; 16d –p = 0.03; 18d –p = 0.03). Glycemia of rats during the wound healing process: (D) diabetic; (DOA) diabetic rats treated with OA. Dashed line indicates the mean of glycemia in control rats. (TIF) [file pone.0165115.s001.tif]
