# Supplementary material for: Oral Administration of Linoleic Acid Induces New Vessel Formation and Improves Skin Wound Healing in Diabetic Rats
Source: PLoS One. 2016 Oct 20;11(10):e0165115. doi: 10.1371/journal.pone.0165115 (PMC5072690; doi:10.1371/journal.pone.0165115)
Supplement: S1 Table — (DOCX) [file pone.0165115.s002.docx]

**S1 Table**. **Primer sequences**

| Gene | Primer sequence |
| --- | --- |
|  | Fo |
| ANGPT-2 | Forward: CTTCAGGTGCTGGTGTCCA  Reverse: GTCACAGTAGGCCTTGACCTC |
| eNOS | Forward: ACCGAGGCAATCTTCGTTCA  Reverse: GTGAAGAGTTCTGGGGGCTC |
| F4/80 | Forward: TTCTGCCATCCTTCACGGAG  Reverse: TGGGTCAGGTACAAGATGCG |
| MPO | Forward: CTGGTGCCACTCCAGCGCTC  Reverse: CCACAGGGGCTGCAGCTTCC |
| TGF-β | Forward: GGGCTACCATGCCAACTTCT  Reverse: GGTTGTAGAGGGCAAGGACC |
| MIP-1αβ | Forward: CGTGTCTGCCTTCTCTCTCC  Reverse: GTGGGAGGGTCAGAGCCTAT |
| MCP-1 | Forward: TATGCAGGTCTCTGTCACGC  Reverse: GAGTAGCAGCAGGTGAGTGG |
| VEGF | Forward: ATGAAGCCCTGGAGTGCGTG  Reverse: TGGCTTTGGTGAGGTTTGATCC |
| HMBS | Forward: GAGACCATGCAGGCCACCAT  Reverse: TTGGAATGTTCCGGGCAGTG |
